# Lack of soluble fiber drives diet-induced adiposity in mice

**DOI:** 10.1152/ajpgi.00172.2015

**Published:** 2015-07-16

**Authors:** Benoit Chassaing, Jennifer Miles-Brown, Michael Pellizzon, Edward Ulman, Matthew Ricci, Limin Zhang, Andrew D. Patterson, Matam Vijay-Kumar, Andrew T. Gewirtz

**Affiliations:** ^1^Center for Inflammation, Immunity and Infection, Institute for Biomedical Sciences, Georgia State University, Atlanta, Georgia;; ^2^Research Diets, Inc., New Brunswick, New Jersey;; ^3^Departments of Veterinary and Biomedical Sciences, Pennsylvania State University, University Park, Pennsylvania; and; ^4^Departments of Nutritional Sciences & Medicine, Pennsylvania State University, University Park, Pennsylvania

**Keywords:** microbiota, short-chain fatty acids, low-grade inflammation, metabolic syndrome

## Abstract

Diet-induced obesity is often modeled by comparing mice fed high-fat diet (HFD), which is made from purified ingredients, vs. normal chow diet (NCD), which is a low-fat assemblage of relatively unrefined plant and animal products. The mechanism by which HFD promotes adiposity is complex but thought to involve low-grade inflammation and altered gut microbiota. The goal of this study was to investigate the extent to which HFD-induced adiposity is driven by fat content vs. other factors that differentiate HFD vs. NCD. Mice were fed NCD, HFD, or other compositionally defined diets (CDD), designed to mimic NCD and/or explore the role of HFD components. A range of metabolic parameters reflecting low-grade inflammation and adiposity were assayed. Relative to NCD, HFD, and to a lesser, but, nonetheless, significant extent, CDD induced increased adiposity, indicating both lipid content and other aspects of HFD are obesogenic. Moreover, HFD and CDD induced a rapid and marked loss of cecal and colonic mass. Such CDD-induced effects were not affected by adjusting dietary protein levels/types but could be largely eliminated by exchanging insoluble fiber (cellulose) for soluble fiber (inulin). Replacing cellulose with inulin in HFD also protected mice against decreased intestinal mass, hyperphagia, and increased adiposity. Such beneficial effects of inulin were microbiota dependent, correlated with elevated fecal short-chain fatty acid levels analyzed via ^1^H-NMR-based metabolomics and were partially recapitulated by administration of short-chain fatty acid. HFD-induced obesity is strongly promoted by its lack of soluble fiber, which supports microbiota-mediated intestinal tissue homeostasis that prevents inflammation driving obesity and metabolic syndrome.

the dramatic increased incidence of obesity, and its interrelated disorders, underscores the importance of understanding the pathophysiology of this disorder. While broad societal changes in diet are almost certainly central to the obesity epidemic, mechanisms by which changes in diet eventuate in obesity are complex. A commonly used means to model diet-induced obesity (DIO) in rodents is to administer an energy dense high-fat diet (HFD). While consumption of HFD likely results directly in extra calories that are efficiently converted to fat, the mechanisms that perpetuate and sustain continued excess caloric consumption are increasingly viewed to involve low-grade inflammation driven, in part, by gut microbiota (17). Specifically, HFD induces alteration of gut barrier function that results in translocation of lipopolysaccharide across the intestine (5, 19). Such lipopolysaccharide may drive low-grade proinflammatory gene expression that can desensitize signaling by insulin (i.e., insulin resistance) and other satiety signaling pathways. In support of this notion, DIO can be ameliorated in germ-free mice, by antibiotic treatment, or by absence of Toll-like receptor (TLR)-4 (3, 6, 22).

Most published studies, including ours (11, 23), that utilize HFD model of DIO examine differences between animals fed “rodent chow” to those fed HFD. However, these diets differ in a number of ways other than their fat content. Chow (e.g., LabDiet5001) is assembled from a broad array of partially processed and relatively unrefined plant and animal products. The components of chow are not well characterized and likely exhibit batch (lot), seasonal, and vendor-to-vendor variability. In contrast, HFD is a compositionally defined diet (CDD) manufactured from purified ingredients, each containing one main nutrient. Thus, as has been noted in the nutrition literature, studies seeking to define the role of HFD components in DIO ought to compare HFD to CDD that differ only in a limited number of specific components, especially percent fat content. Indeed, herein we report that two different commercial CDD, designed to match chow nutritionally and to be control diets for HFD, drove decreased intestinal mass and increased adiposity relative to mice fed chow. Investigation of the underlying mechanisms revealed that, in contrast to chow, HFD and control CDD failed to support intestinal health, correlating with their lack of soluble fiber. Addition of soluble fiber to CDD restored intestinal health and protected against HFD-induced adiposity. Such beneficial effects of inulin were dependent on the presence of a microbiota and correlated with increased production of short-chain fatty acids (SCFA). Thus we report that use of CDD ought to contain soluble fiber to maintain a healthy intestine, and that supplementation of various diets might further promote intestinal health that can protect against low-grade inflammation that drives adiposity.

## MATERIALS AND METHODS

### 

#### Mice.

Wild-type C57BL/6 mice were purchased from Jackson Laboratories. All mice were then housed at Georgia State University (Atlanta, GA) under institutionally approved protocols (Institutional Animal Care and Use Committee no. A14033). Mice were fed Purina rodent chow (cat. no. 5001) from LabDiets, unless another diet is specified.

#### Diets.

Diets were procured form Research Diets (New Brunswick, NJ) or Harlan (Indianapolis, IN). The composition of all diets used in this study was detailed in Tables 1 and 2. Fiber was added on a similar per kilocalorie basis in low- and high-fat diet formulas, and inulin was considered as 1.0 kcal/g, replacing an equal amount of kilocalories from starch (or maltodextrin in formulas with 60 kcal% fat). We considered cellulose to provide no calories.

**Table 1.** Purified diets used in the study

	D12450B	D12492	D13081101	D13081104	D11112212	D11112213	D11112208	D11112209	D11112210	D11112211	D13081102	D13081103	D14061501	D14061502
Protein source	Casein	Casein	Casein	Casein	Soy	Soy	Casein	Soy	Casein	Casein	Casein	Casein	Casein	Casein
Fiber source	Cellulose	Cellulose	Inulin	Inulin	Cellulose	Inulin	Mixed	Mixed	Cellulose	Inulin	Inulin	Inulin	Cellulose	Inulin
Kilocalories% fat	10	60	10	60	15	15	15	15	15	15	10	10	45	45
Protein, kcal%	20	20	20	20	20	20	20	20	20	20	20	20	20	20
Carbohydrate, kcal%	70	20	69	19	65	60	62	62	65	60	68	65	35	33
Fat, kcal%	10	60	10	60	15	15	15	15	15	15	10	10	45	45
Total	100	100	100	100	100	100	100	100	100	100	100	100	100	100
Kilocalories/gram	3.8	5.2	3.9	5.3	3.5	3.6	3.5	3.5	3.5	3.6	3.8	3.5	4.5	4.6
Ingredient														
Casein (4 kcal/g), g	200	200	200	200	0	0	200	0	200	200	200	200	200	200
Soy protein (4 kcal/g), g	0	0	0	0	200	200	0	200	0	0	0	0	0	0
dl-Methionine (4 kcal/g), g	0	0	0	0	3	3	0	3	0	0	0	0	0	0
l-Cystine (4 kcal/g), g	3	3	3	3	0	0	3	0	3	3	3	3	3	3
Corn starch (4 kcal/g), g	315	0	302.5	0	387	337	362	362	387	337	290	265	72.8	72.8
Maltodextrin 10 (4 kcal/g), g	35	125	35	112.5	110	110	110	110	110	110	35	35	100	75
Sucrose (4 kcal/g), g	350	68.8	350	68.8	150	150	150	150	150	150	350	350	172.8	172.8
Cellulose (0 kcal/g), g	50	50	0	0	200	0	100	100	200	0	0	0	100	0
Inulin (1 kcal/g), g	0	0	50	50	0	200	100	100	0	200	100	200	0	100
Soybean oil (9 kcal/g), g	25	25	25	25	70	70	70	70	70	70	25	25	25	25
Lard (9 kcal/g), g	20	245	20	245	0	0	0	0	0	0	20	20	177.5	177.5
RDI mineral mix (0 kcal/g), g	45	45	45	45	45	45	45	45	45	45	45	45	45	45
Vitamin mix, V10001 (4 kcal/g), g	10	10	10	10	10	10	10	10	10	10	10	10	10	10
Choline bitartrate (0 kcal/g), g	2	2	2	2	2	2	2	2	2	2	2	2	2	2
Total grams	1,055.05	773.8	1,042.5	761.30	1,177.00	1,127.00	1,152.00	1,152.00	1,177.00	1,127.00	1,080.00	1,155.00	908.10	883.10
Total kilocalories	4,057	4,057	4,057	4,057	4,070	4,070	4,070	4,070	4,070	4,070	4,057	4,057	4,057	4,057

RDI, recommended daily intake.

**Table 2.** Chow-based diets used in the study

	C11000	C13062905	C13062906	C13062903	C13062904	C13887	C13889
Protein source	Multiple	Multiple	Multiple	Multiple	Multiple	Multiple + added Casein	Multiple + added Soy
Fiber source	Multiple	Multiple + added cellulose	Multiple + added cellulose	Multiple + added inulin	Multiple + added inulin	Multiple	Multiple
Ingredient							
Purina 5001, g	1,000	900	800	900	800	850	850
Casein, g	0	0	0	0	0	150	0
Soy protein, g	0	0	0	0	0	0	150
Cellulose, g	0	100	200	0	0	0	0
Inulin, g	0	0	0	100	200	0	0
FD&C red dye 40, g	0	0.15	0.075	0	0	0	0
FD&C blue dye 1, g	0	0	0	0.15	0.075	0	0
Total	1,000	1,000.15	1,000.075	1,000.15	1,000.075	1,000	1,000

FD&C, Food, Drug & Cosmetic Colors.

#### Mice treatment.

Mice were fed with “normal” chow diet (NCD) of specified CDD for a period of 2 days to 12 wk. NCDs were autoclaved for experiments using germ-free mice. Body weights were measured every week and are expressed as a percentage gain compared with the initial body weight (*day 0*), defined as 100%. At the end of diet treatment, mice were fasted for 5 h. Mice were then euthanized, and colon length, colon weight, spleen weight, and adipose weight were measured. Organs were collected for downstream analysis.

#### Food intake measurement.

Groups of mice were placed in a clean cage with a known amount of food. Twenty-four hours later, the amount of remaining food was measured with the difference viewed as food intake per 24 h.

#### Antibiotic treatment.

Mice were placed on broad-spectrum antibiotics ampicillin (1.0 g/l) and neomycin (0.5 g/l) in drinking water for 4 wk (23). As ampicillin and neomycin are poorly absorbed, such treatment primarily affects only intestinal microbiota without direct systemic effects (15).

#### SCFA treatment.

Mice were put on drinking water supplemented with a mixture of SCFA (67.5 mM sodium acetate, 40 mM sodium butyrate, and 25.9 mM sodium propionate) for 21 days (21).

#### Germ-free experiments.

Germ-free C57BL/6 mice were kept under germ-free conditions in a Park Bioservices isolator in our germ-free mice facility. After 4 wk of diet feeding, mice were fasted for 5 h and then removed from the isolator to be euthanized immediately. Samples were collected as previously described.

#### NMR spectroscopy.

All ^1^H NMR spectra of fecal extracts were recorded at 298 K using a Bruker Avance III 600 MHz NMR spectrometer (operating at 600.08 MHz for proton, Bruker Biospin), equipped with an inverse cryogenic probe. A one-dimensional NMR spectrum was acquired for each of all samples, employing the first increment of NOESY pulse sequence (recycle delay-90°-*t*_1_-90°-*t*_m_-90°-acquisition) with a spoil gradient for water presaturation. The recycle delay of 2 s, *t*_1_ of 4 μs, and the mixing time (*t*_m_) of 100 ms were set. The 90° pulse length was adjusted to 10 μs. A total of 64 scans were collected into 32k data points for each spectrum with a spectral width of 20 ppm. For resonance assignment purposes, a series of two-dimensional NMR experiments (^1^H−^1^H correlation spectroscopy, ^1^H−^1^H total correlation spectroscopy, ^1^H−^13^C heteronuclear single quantum correlation spectroscopy, and ^1^H−^13^C heteronuclear multiple bond correlation spectroscopy) were carried out on the selected samples.

#### Spectral data processing and multivariate data analysis.

^1^H NMR spectra were corrected manually for phase and baseline distortions, and spectral region δ 0.5–9.5 was integrated into regions with equal width of 0.004 ppm (2.4 Hz) using AMIX software package (V3.8, Bruker-Biospin). Regions distorted were discarded. These regions are δ 4.45–5.20 for imperfect water saturation in both cecal and fecal extracts, and δ 1.15–1.23 and δ 3.62–3.69 for ethanol contaminations during the mouse dissection process. Each bucketed region was then normalized to the total sum of the spectral integrals to compensate for the overall concentration differences before statistical data analysis.

Multivariate data analysis was carried out with the SIMCAP+ software (version 13.0, Umetrics, Sweden). Principal component analysis was initially carried out on NMR data to generate an overview. Orthogonal projection to latent structure with discriminant analysis (OPLS-DA) was subsequently conducted using NMR data. The OPLS-DA models were validated using a sevenfold cross-validation method, and the quality of the model was described by the parameters R^2^X and Q^2^ values. To facilitate interpretation of the results, back-transformation (13) of the loadings generated from the OPLS-DA was performed before generating the loadings plots, which were color-coded with the Pearson linear correlation coefficients of variables (or metabolites) using an in-house developed script for MATLAB (The Mathworks, Natwick, MA). The color-coded correlation coefficient indicates the significance of the metabolite contribution to the class separation, with a “hot” color (e.g., red) being more significant than a “cold” color (e.g., blue). In this study, a cutoff value of |*r*| > 0.707 (*r* > 0.707 and *r* < −0.707) was chosen for correlation coefficient as significant based on the discrimination significance (*P* ≤ 0.05).

#### Statistical analysis.

Significance was determined using one-way ANOVA corrected for multiple comparisons with a Bonferroni test (GraphPadPrism software, version 6.01). Differences were noted as significant at *P* ≤ 0.05.

## RESULTS

### 

#### CDD are obesogenic and induce loss of intestinal mass.

**Fig. 1.**
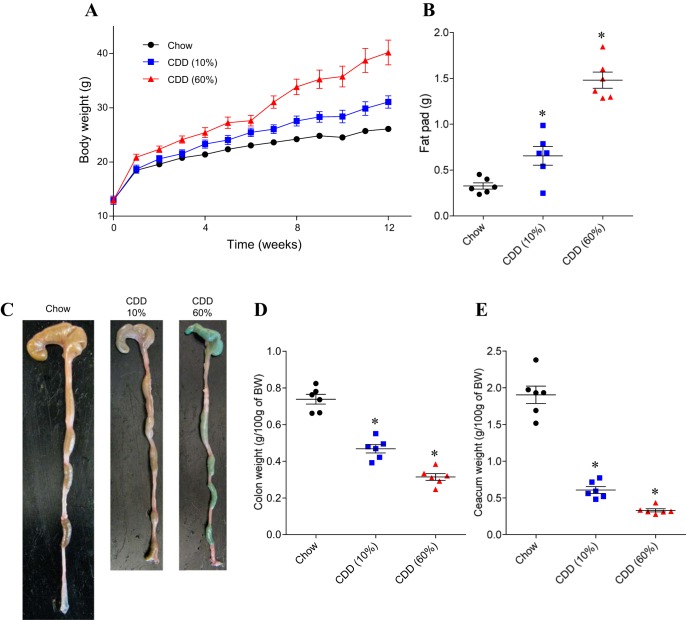
Compositionally defined diets (CDD) are obesogenic and induce loss of intestinal mass. C57Bl/6 mice were maintained on the “normal” chow diet (NCD), or, at 4–5 wk of age, switched to the specified CDD for 12 wk (with 10 or 60% fat). *A*: body weight (BW) over time. *B*: epididymal fat pad weight. *C*: gross pictures of gut morphology. *D*: colon weight. *E*: cecum weight. Values are means ± SE of *N* = 6 mice per group. Significance was determined by Student's *t*-test. *P < 0.05.

C57Bl/6 mice, in all cases herein fed ad libitum, were maintained on a NCD through the postweaning period (i.e., until 6 wk of age) so as to allow their mucosal immune system to mature and gut microbiota composition to stabilize (10, 14). Mice were then switched to one of two CDD, which are assembled from purified quality-controlled ingredients. One CDD contained 60% fat, i.e., HFD (HFD or CDD-60%), and has been widely utilized in research to promote obesity, while the other CDD contained 10% fat (CDD-10%) and can be viewed as an appropriate control diet for the HFD in that it is made from the same ingredients, albeit in different proportions and, moreover, is thought to be similar to chow in terms of its basic nutrient/calorie content. Relative to NCD-fed mice, mice fed HFD exhibited marked weight gain (Fig. 1*A*) that corresponded with development of epididymal fat pads about fourfold larger than that of NCD-fed animals (Fig. 1*B*). An intermediate result was observed with mice fed a CDD composed of 10% fat (CDD-10%). (Fig. 1, *A* and *B*). This pattern of results was consistently seen in multiple experiments and when both CDD were purchased from different vendor (data not shown). These results confirm that fat content is a key driver of the DIO phenotype, but also indicate that other differences between HFD and NCD contribute to HFD's promotion of obesity. Obesity is associated with, and promoted by, low-grade chronic inflammation. A correlate of low-grade intestinal inflammation is shortening of the colon, which is often accompanied by loss of cecal and colonic mass (24). Indeed, a striking consequence of feeding mice either HFD or control CDD was a dramatic loss of cecal and colonic tissue mass. Specifically, relative to chow-fed mice, the intestine of CDD-fed mice appeared thin and atrophied (Fig. 1*C*), which correlated with a shortening of the colon and loss of cecal and colonic mass that could be quantitated by weighing cecum (with contents) and colon (without contents) (Fig. 1, *D* and *E*). We reasoned that such gross effects on the intestine by CDD might be pivotal to their promotion of adiposity and, hence, sought to understand their cause.

#### CDD-induced changes are rapid and involve gut bacteria.

**Fig. 2.**
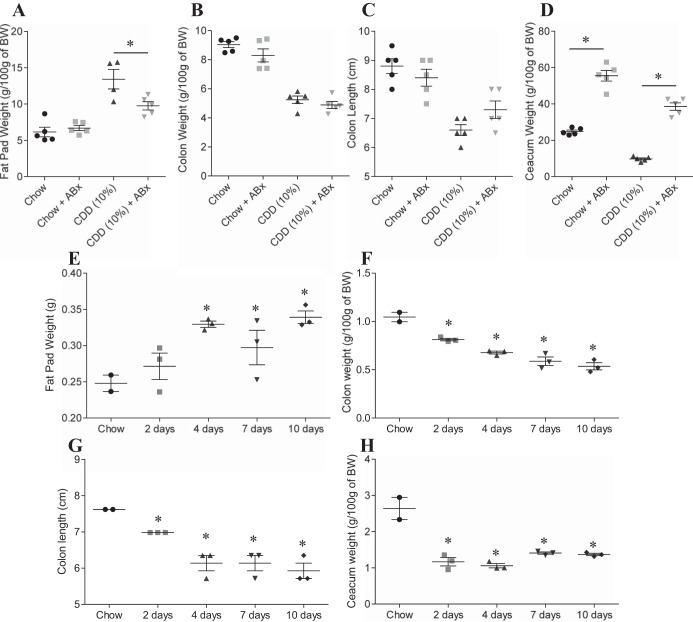
Microbiota involvement in protection of weight gain and intestinal mass loss induced by CDD. *A–D*: C57Bl/6 mice were maintained on the NCD, or, at 4–5 wk of age, switched to the specified diet and treated with ampicillin (1.0 g/l) and neomycin (0.5 g/l) in drinking water for 4 wk. *A*: epididymal fat pad weight. *B*: colon weight. *C*: colon length. *D*: cecum weight. *E–H*: C57Bl/6 mice were maintained on the NCD, or, at 4–5 wk of age, switched to the specified diet for 2–10 days. *E*: epididymal fat pad weight. *F*: colon weight. *G*: colon length. *H*: cecum weight. Values are means ± SE of *N* = 5 (*A–D*) or 3 (*E–H*) mice per group. Significance was determined by Student *t*-test. *P < 0.05. ABx, antibiotics.

**Fig. 3.**
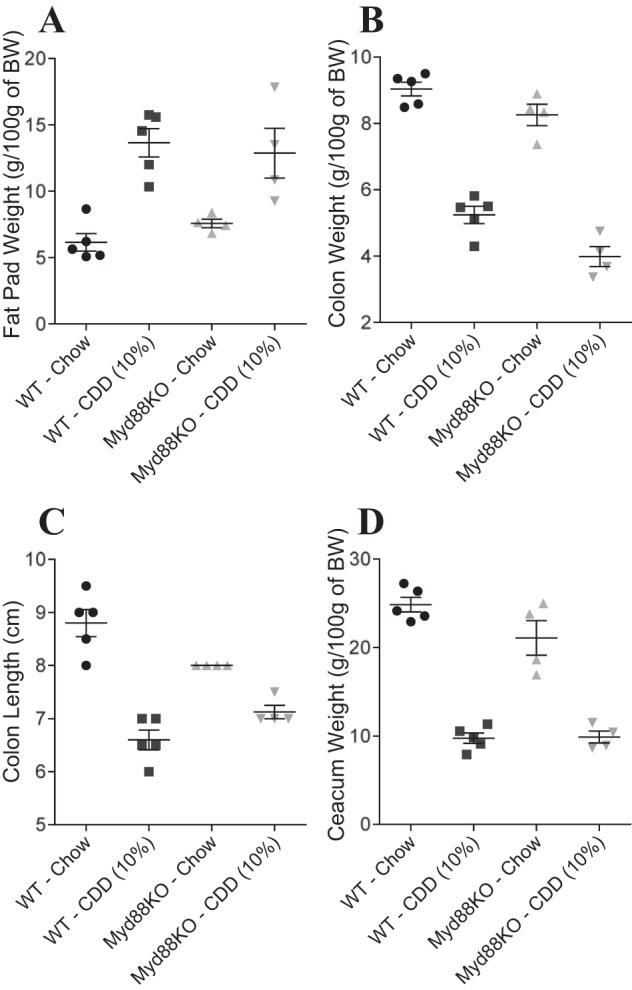
Myd88 involvement in weight gain and intestinal mass loss induced by CDD. C57Bl/6 WT or Myd88 knockout (KO) mice were maintained on the NCD, or, at 4–5 wk of age, switched to the specified diet for 12 wk. *A*: epididymal fat pad weight. *B*: colon weight. *C*: colon length. *D*: cecum weight. Values are means ± SE of *N* = 5 mice per group.

HFD-induced obesity is thought to involve interactions between the gut microbiota and host innate immune system that promotes low-grade inflammation. Consequently, the extent to which obesity is induced by HFD is reduced by antibiotic treatment (6). Analogously, the adiposity induced by low fat (10%) CDD-10% was significantly reduced by antibiotic treatment (Fig. 2*A*). Moreover, such amelioration of CDD-induced adiposity by antibiotic treatment was accompanied by a partial restoration of cecal and colonic tissue (Fig. 2, *B–D*). We hypothesized that these results might reflect that the CDD were increasing bacterial activation of MyD88 signaling, which is known to mediate a considerable portion of TLR signaling. However, the effects of CDD were only modestly reduced in MyD88-deficient mice, suggesting this pathway did not play a major role in mediating CDD's effect on the intestine and adipose tissue (Fig. 3). Next, we determined the time course with which CDD results in loss of intestinal mass. We observed that 2 days of feeding of CDD was sufficient to result in a dramatic loss of cecal mass (Fig. 2*H*) and a significant loss of colon mass/length, which continued to decline over the next several days (Fig. 2, *F* and *G*) and by 10 days was similar in magnitude to that seen in experiments where the diet was maintained for several weeks. A significant increase in adiposity was also observed over this relatively short time course (Fig. 2*E*). Together, these results suggest that CDD may alter gross intestinal morphology in a rapid manner that promotes microbiota-dependent, low-grade inflammation that promotes adiposity.

#### Dietary fiber content, but not protein, alters gut morphology alterations induced by CDD.

**Fig. 4.**
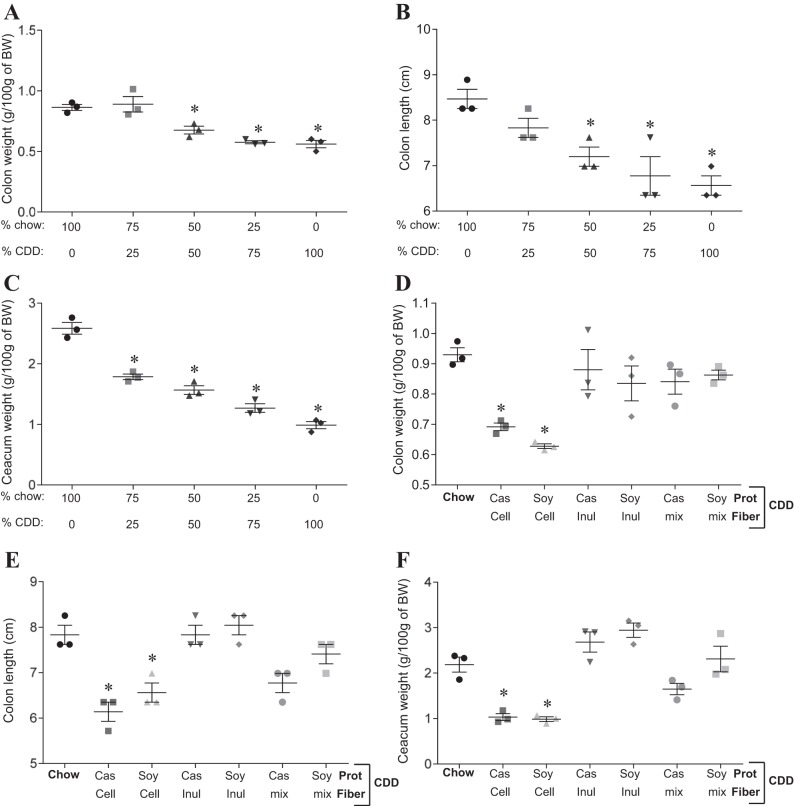
Fiber content, but not protein (Prot) content, alters gut morphology induced by CDD. C57Bl/6 mice were maintained on the NCD, or, at 4–5 wk of age, switched to the specified diet for 4 wk. *A–C*, percentage of diet composed of chow and CDD is shown. *D–F*: Prot and fiber components of the CDD are shown. *A* and *D*: colon weight. *B* and *E*: colon length. *C* and *F*: cecum weight. Cell, cellulose; Inul, inulin; Cas, casein. Values are means ± SE of *N* = 3 mice per group. Significance was determined by Student *t*-test. *P < 0.05.

**Fig. 5.**
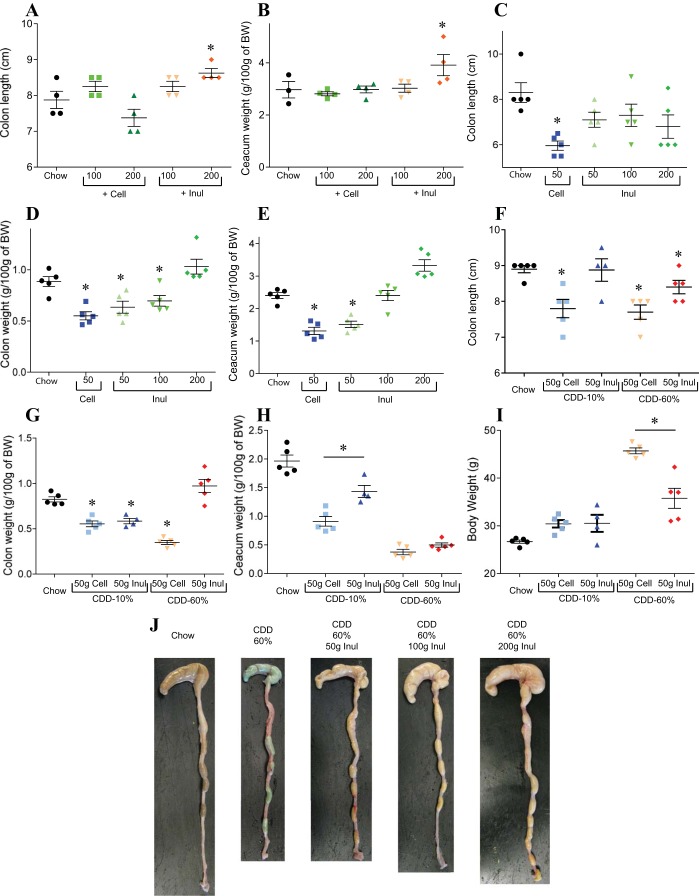
Soluble fiber-supplemented CDD protect from gut morphology alterations. C57Bl/6 mice were maintained on the NCD, or, at 4–5 wk of age, switched to the specified diet for 4 wk. *A* and *B*: chow plus Cell or Inul. *C–E*: CDD plus Cell or Inul. *F–I*: chow and CDD with 10% and 60% fat plus Cell or Inul. *A*, *C*, and *F*: colon length. *B*, *E*, and *H*: cecum weight. *C* and *F*: colon length. *D* and *G*: colon weight. *I*: BW. *J*: gross pictures of gut morphology. Values are means ± SE of *N* = 4 (*A* and *B*) or 5 (*C–J*) mice per group. Significance was determined by Student *t*-test. *P < 0.05.

**Fig. 6.**
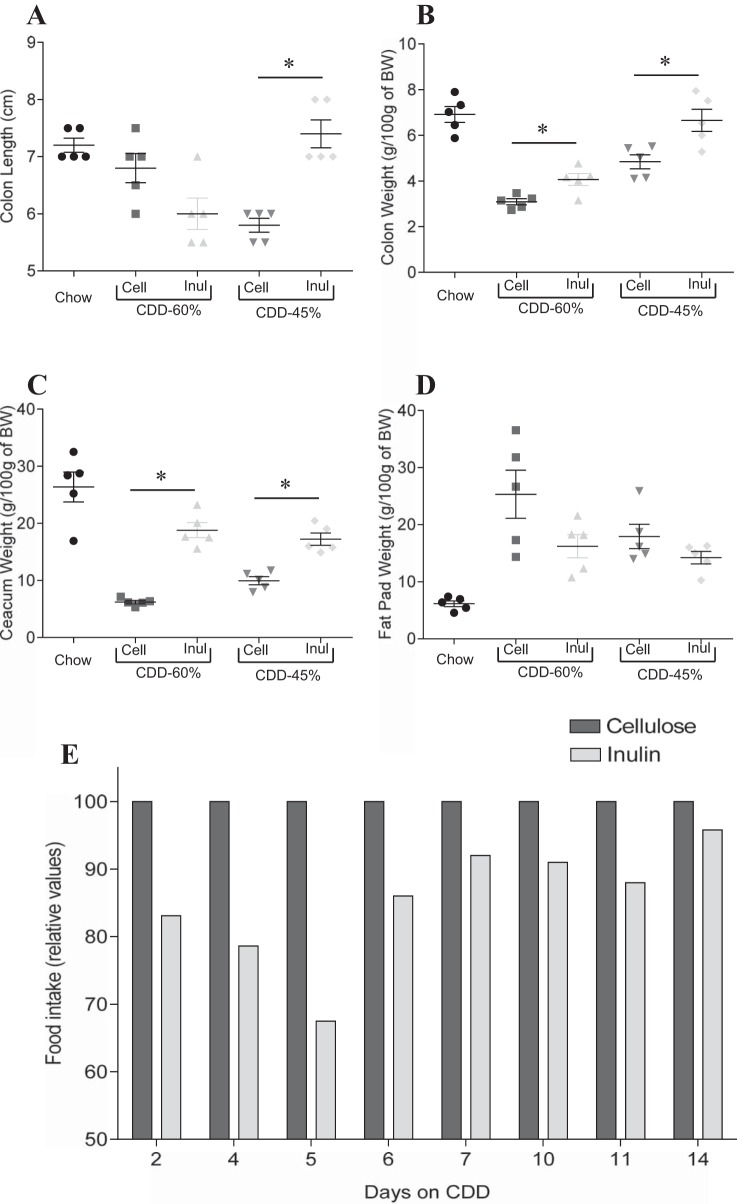
Soluble fiber-supplemented CDD protect from gut morphology alterations. C57Bl/6 mice were maintained on the NCD, or, at 4–5 wk of age, switched to the specified diet for 6 wk. *A*: colon length. *B*: colon weight. *C*: cecum weight. *D*: epididymal fat pad weight. *E*: food intake. Values are means ± SE of *N* = 5 mice per group. Significance was determined by Student *t*-test. *P < 0.05.

In light of our hypothesis that low-grade inflammation plays a key role in DIO, and that a healthy gut is necessary to protect the host against inflammation, we sought to better understand why CDD might result in loss of intestinal mass. First, to get a better sense of the extent to which this phenotype was driven by absence of a gut-maintaining component of NCD or presence of a gut-destroying component of CDD, we mixed the diets at a range of ratios of dietary protein or fibers. The loss of intestinal mass phenotype was proportional to the percentage of the diet composed of CDD, a result consistent with the possibility that the phenotype resulted from loss of a nutrient that was limiting in chow, or that it was a dose-dependent consequence of a component of CDD (Fig. 4, *A–C*). We first considered the possibility that the protein component of the CDD, namely purified animal protein casein, might be playing a role in the CDD-induced phenotype. Hence, we formulated diets in which the purified protein in CDD, casein, was replaced with plant-derived soy protein (plant protein source in NCD). This change did not significantly impact the CDD-induced loss of intestinal mass or its associated metabolic phenotypes (Fig. 4, *D–F*). Nor did addition of casein or soy to NCD alter these parameters relative to a NCD alone, thus arguing against a role for protein type and content (data not shown). Next, we considered the role of the fiber, which is present in NCD, albeit not in a defined amount. HFD, and therefore its matched low-fat control CDD, contain the insoluble fiber (resistant to bacterial fermentation) cellulose as a sole source of fiber. Exchanging cellulose for the soluble fiber (readily undergo bacterial fermentation) inulin largely reversed the deleterious effects of the CDD on the intestine (Fig. 5, *F–I*). This was true, regardless of whether the CDD had a protein source of casein or soy protein. Furthermore, mixing cellulose and a CDD containing both inulin and cellulose did not result in loss of gut mass, suggesting this phenotype may result from CDD's detriment of soluble fiber rather than the presence of cellulose per se. Next, we administered diets designed to investigate whether cellulose might have detrimental effects or if these results reflected that inulin was promoting gut health, as previously suggested (1, 7, 18). In support of this notion, addition of cellulose to chow resulted in only very modest loss of gut mass and did so only at relatively high concentrations (4× the fiber typically in CDD) (Fig. 5, *A* and *B*). Rather, in CDD, levels of inulin correlated well with maintenance of gut mass (Fig. 5, *C–E*), could correct loss of gut mass induced by CDD-10% or CDD-60% (Fig. 5, *G–J*), and addition of inulin to NCD boosted the level of intestinal mass relative to NCD alone (Fig. 5, *A* and *B*). The restoration of gut mass achieved by exchange of cellulose for inulin correlated with amelioration in body weight gain and adiposity induced by HFD (Fig. 6*D*). We hypothesized that the inulin's reduction in HFD would correlate with lower food consumption. However, the physical nature of the HFD makes this measure very difficult, as the diet crumbles and cannot be easily separated from cage bedding (we sought to avoid use of metabolic cages, which can be very stressful to mice and might impact feeding behavior). Hence, we designed a CDD with 45% fat (CDD-45%), which does not preclude food consumption measurements and contained cellulose or inulin as a fiber source. Analogous to the other CDD, inulin preserved gut mass and reduced adiposity relative to mice fed CDD-45% that contained cellulose (Fig. 6, *A–D*). Moreover, such reduction in adiposity in mice fed inulin CDD-45% correlated with reduced food consumption (Fig. 6*E*). Together, these results suggest that soluble fiber may be a limiting nutrient in NCD and that its complete absence in CDD contributes to DIO. Moreover, soluble fiber may be a means of maintaining gut health and preventing diseases driven by low-grade inflammation.

#### SCFA involvement in soluble fiber protection of gut morphology alterations.

**Fig. 7.**
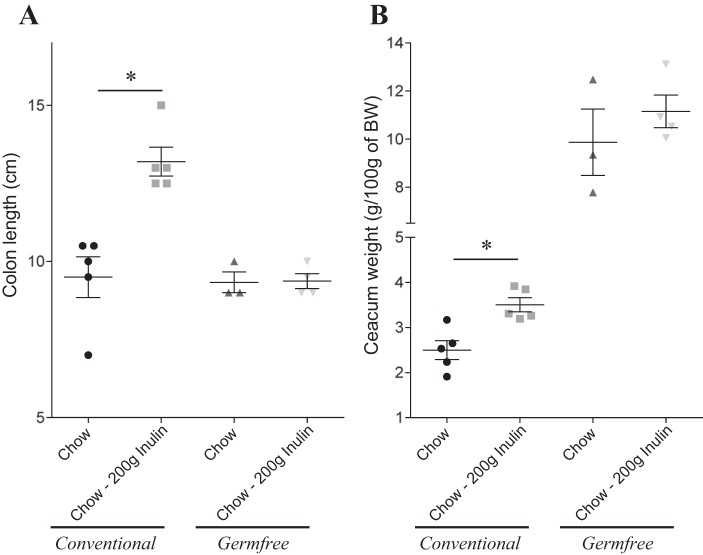
Microbiota involvement in soluble fiber protection of gut morphology alterations. Conventional and germ-free C57Bl/6 mice were maintained on autoclaved NCD, or, at 4–5 wk of age, switched to the specified autoclaved diet for 4 wk. *A*: colon length. *B*: cecum weight. Values are means ± SE of *N* = 3–5 mice per group. Significance was determined by Student *t*-test. *P < 0.05.

**Fig. 8.**
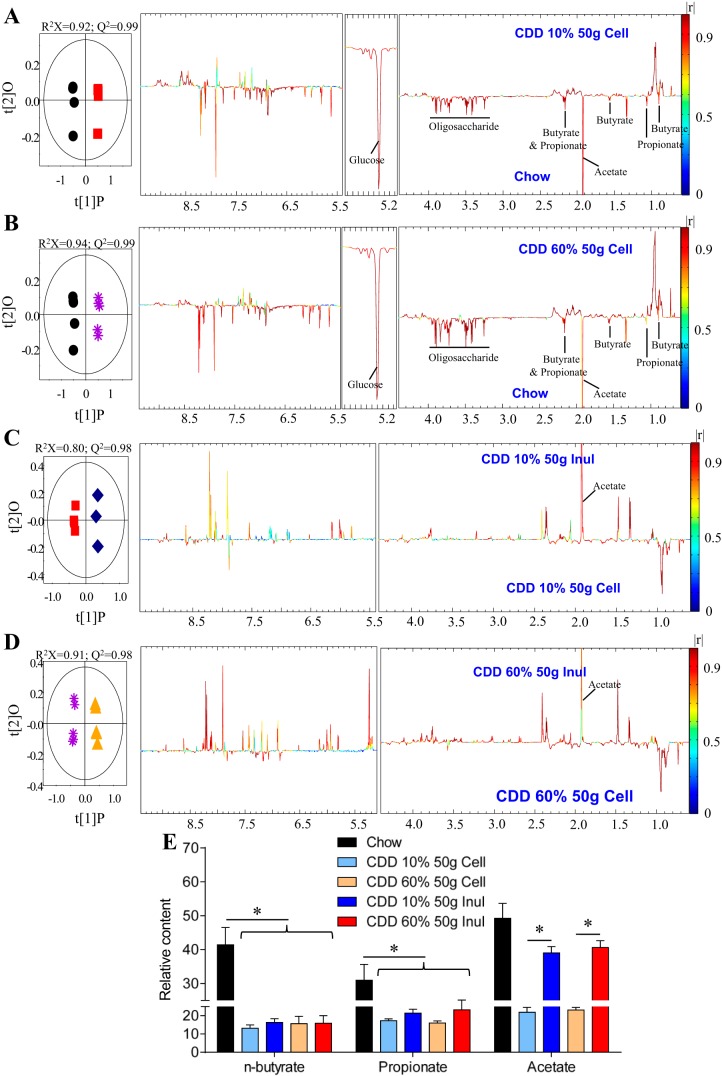
NMR-based metabolomics reveal short-chain fatty acid (SCFA) involvement in soluble fiber-induced protection of gut morphology alterations. *A–D*: orthogonal projection to latent structure with discriminant analysis score plot (*left*) and correlation coefficient loading plot (*right*) showing the discrimination between ^1^H NMR spectra of fecal contents from mice fed with the indicated diet for 4 wk. *A*: NCD vs. CDD-10%. *B*: NCD vs. CDD-60%. *C*: CDD-10% vs. CDD-10% + 200 g Inul. *D*: CDD-60% vs. CDD-60% + 200 g Inul. *E*: relative content of *n*-butyrate, propionate, and acetate in mice fed with the indicated diet for 4 wk. Values are means ± SE of *N* = 5 mice per group. Significance was determined by Student *t*-test. *P < 0.05.

**Fig. 9.**
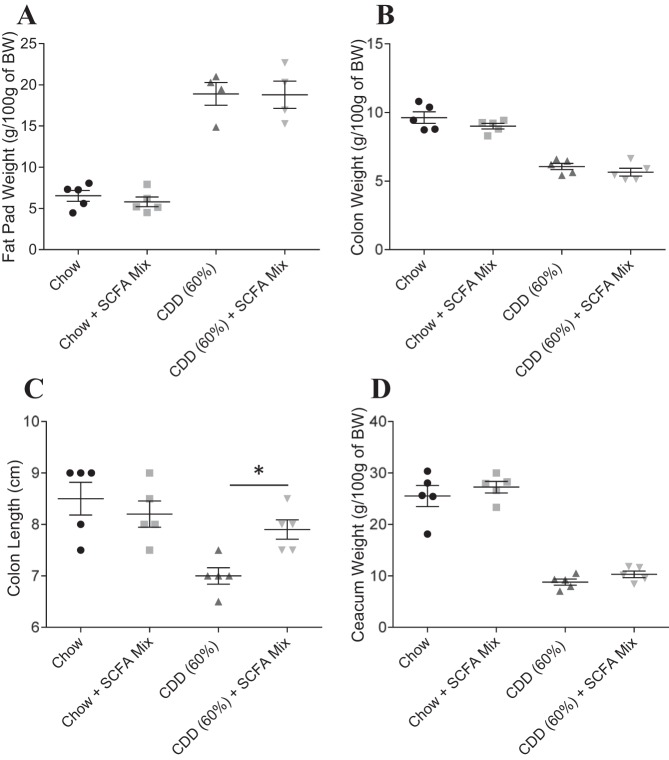
SCFA involvement in soluble fiber protection of gut morphology alterations. C57Bl/6 mice were maintained on the NCD, or, at 4–5 wk of age, switched to the specified diet and treated with SCFA mix (67.5 mM sodium acetate, 40 mM sodium butyrate, and 25.9 mM sodium propionate) in drinking water for 3 wk. A: epididymal fat pad weight. *B*: colon weight. *C*: colon length. *D*: cecum weight. Values are means ± SE of *N* = 5 mice per group. Significance was determined by Student *t*-test. *P < 0.05.

We next sought to better understand the mechanism by which inulin promotes gut health. First, we used a germ-free approach to investigate microbiota involvement. However, unlike chow, CDD cannot be autoclaved, and efforts to sterilize CDD by irradiation at typical doses (10–20 kGy) were not successfully achieved (i.e., germ-free mice fed an irradiated CDD developed high level of cultivable fecal bacteria; data not shown). Hence, we administered sterilized (i.e., autoclaved) inulin-supplemented NCD to conventional and germ-free mice and investigated if lack of a microbiota prevented inulin's ability to boost gut mass. We observed that inulin's ability to promote colon and cecum mass was completely absent in germ-free mice, indicating its promotion of gut health is likely microbiota dependent (Fig. 7). Microbiota-mediated production of SCFA is thought to be an important energy source for gut epithelia and which bacteria can readily generate from soluble but not insoluble fiber. Hence, we next measured levels of fecal SCFA via NMR, and we observed that, relative to NCD-fed mice, mice fed CDD, both CDD-10% and HFD-60%, exhibited reduced levels of butyrate, acetate, and propionate (Fig. 8, *A*, *B*, *E*). Exchanging the cellulose in these diets for inulin boosted SCFA levels (Fig. 8, *C–E*), supporting the notion that inulin promotes gut health via microbiota-mediated production of SCFA that protect against inflammation. To further examine this possibility, we administered SCFA to the drinking water of CDD-fed mice. Such SCFA treatment did not recapitulate all of inulin's beneficial effect, but attenuated CDD-induced colonic shortening, indicating partial protection against CDD-induced low-grade inflammation (Fig. 9). Together, these results indicate lack of microbiota-produced soluble fiber metabolites is pivotal to the maintenance of a healthy mouse intestine and suggest that SCFA production is one specific class of metabolites involved in this process.

## DISCUSSION

Societal changes in food production and diet are presumed to be major contributors to the epidemic of obesity and its associated diseases. In particular, the increased availability and consumption of foods rich in fat and calories is thought to be a major factor underlying the epidemic of obesity and its related diseases; i.e., metabolic syndrome. In support of this notion, ad libitum feeding of a HFD to mice rapidly increases and recapitulates and/or promotes many features of metabolic syndrome. Consequently, administration of HFD has been widely used to investigate the pathophysiology and potential treatments of metabolic syndrome. Such studies indicate that HFD-induced metabolic disease may be driven, in part, by altered host-microbiota interactions that drive low-grade inflammation (8). Yet, how the HFD alters such events remains largely unknown. Even the relative role of the HFD's fat content vs. the numerous other ways by which this diet differs from the NCD to which it is typically experimentally compared have not been well defined. Herein, we observed that, while fat content is an important determinant of the extent to which HFD promotes adiposity, other determinants of this diet are pivotal in driving low-grade inflammation and, consequently, likely contribute to the HFD-induced phenotype. Specifically, we observed that, among mice fed NCD vs. a range of CDD, lack of soluble fiber correlated with cecal and colonic atrophy that resulted in microbiota-dependent promotion of adiposity. The notion of diet-induced, microbiota-dependent increase in adiposity is in accord with the more general hypothesis that a broad range of genetic and nongenetic factors that result in disturbance of the microbiota can promote metabolic disease (9–11). Consequently, that addition of exogenous soluble fiber (i.e., inulin) protected against both gut atrophy and HFD-induced adiposity suggests that increasing dietary soluble fiber intake may have broad applicability in ameliorating metabolic disease.

Dietary fiber intake, which has long been appreciated as an effective means to treat and prevent constipation, is increasingly recognized for its association with metabolic health. Consequently, US Department of Agriculture dietary guidelines encourage consumption of foods rich in fibers, but do not distinguish between soluble and insoluble fibers. Yet our results herein that a soluble fiber, namely inulin, but not an insoluble one, namely cellulose, promoted bowel mass and attenuated the obesogenic effects of a HFD suggest a major distinction in the ability of such fibers to promote health. The ability of inulin to suppress adiposity by CDD diets high in fat content correlated with reduced food intake, which seems highly likely to have been a factor in reducing adiposity. That such ability was observed relative to CDD containing cellulose argues against the notion that such actions reflect simple addition of dietary bulk, but, rather, suggest a key difference in how such fibers are metabolized. Gut microbiota readily metabolize soluble fiber into SCFA, notably butyrate, which is known to serve as a major fuel source for colonic epithelia and have an array of anti-inflammatory properties, including promotion of regulatory T cells (2, 16, 20). Accordingly, we and others have observed near complete absence of SCFAs in germ-free mice and, herein, observed that the ability of inulin to increase gut mass was absolutely dependent on the presence of a gut microbiota, suggesting that SCFA production might contribute to inulin's beneficial effects observed in our model. SCFA-mediated nourishment of the epithelium can be envisaged to result in enhanced barrier function that might reduce exposure of immune cells to bacterial products that would drive inflammatory gene expression and, consequently, phenotypes associated with gut inflammation. However, that addition of SCFA, following a regimen reported by others to support gut regulatory T-cell homeostasis (2, 16, 20), only partially recapitulated the gut protective effects exhibited by inulin, suggesting that SCFA production may be but one mechanism by microbiota metabolism of inulin protection against gut atrophy (21).

A pivotal observation that drove this study is that, relative to NCD, the ill-defined conglomerate of food scraps (grains and animal by-products) that most rodents in biomedical research are fed, CDD (with cellulose) assembled from characterized ingredients results in rapid gut atrophy, which seems likely to play a role in the obesogenic potential of such diets. Specifically, considering that intestinal cell proliferation protects the gut from a range of inflammatory challenges and HFD-induced obesity involves gut barrier dysfunction and subsequent microbiota activation of proinflammatory gene expression, we envisage that such gut atrophy results in increased bacterial induction of low-grade inflammation that can promote adiposity (10, 23). That some indexes of CDD-induced low-grade inflammation, particularly colon shortening, were ameliorated in MyD88-deficient mice suggests a role for TLR-mediated detection of bacterial products in driving such low-grade inflammation, which is in accord with work of Cani and colleagues that TLR-4 drives HFD-induced metabolic syndrome (5). Yet, that such amelioration was only partial indicates that other pathways are also likely involved. Given that a large portion of gene expression induced by TLR agonists is in fact MyD88-independent (4), additional work will be needed to define other signaling pathways that contribute to CDD-induced phenotypes. Moreover, the state of low-grade inflammation induced by CDD is not itself well defined, in that CDD induced only very modest increases in myeloperoxidase and, moreover, did not associate with clear increases in fecal lipocalin-2, which we have found can reliably mark low-grade inflammation in chow-fed mice (12). We hypothesize that this reflects that fecal lipocalin-2 is largely produced by epithelial cells, thus obviating its utility as a marker in states wherein inflammation may have resulted from gut atrophy. Hence, in future work, it will be important to better define the low-grade inflammatory state that results from CDD feeding and to determine how this state influences, and is influenced by, gut microbiota.

**Fig. 10.**
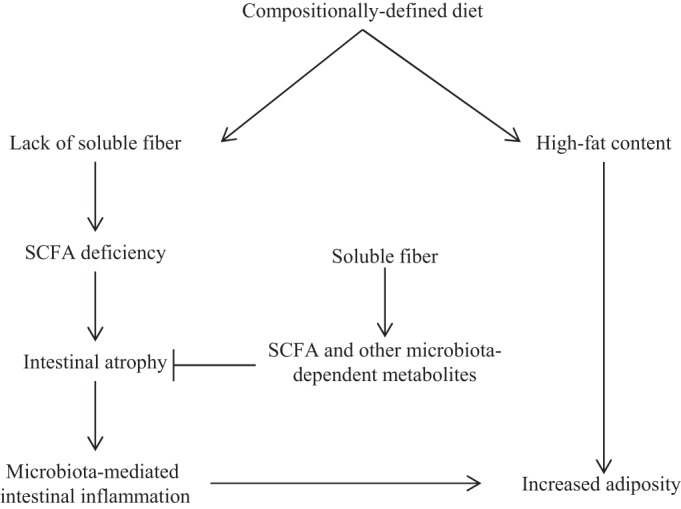
Proposed mechanism.

In any case, that such phenotypes could be largely prevented by addition of inulin suggests the CDD induces gut atrophy as a result of its lack of soluble fiber. However, it is important to note that, at present, we cannot rule out the possibility that the CDD causes gut atrophy by another mechanism and that inulin simply protects against this phenotype by a mechanism not related to its underlying cause (Fig. 10). Regardless, the ability of inulin to promote gut mass when added to a wide variety of diets may make it an important ingredient in biomedical research. Specifically, the ill-defined nature of NCD, its consequent, inherent variability (vendors and different facilities/countries), and, perhaps most importantly, the inability to precisely manipulate its components greatly hinder its utility in a broad range of experimental approaches, especially those related to macro/micronutrient metabolism and host metabolic phenotype. Yet the dramatic deleterious effects of commonly used CDD on gut health, irrespective of fat content, suggest that such diets are not a good alternative to NCD. In contrast, as shown herein, CDD containing inulin remedy this concern and may serve as a good choice of control diet that would be a better mimic of NCD but yet retain tractability.

In conclusion, supplementation of a broad range of mouse diets with inulin promotes gut mass and protects against DIO by a microbiota-dependent mechanism, albeit one whose understanding is far from complete. If our observations were to prove applicable to humans, it would suggest encouraging consumption of foods with high soluble fiber content may be a means to combat the epidemic of metabolic disease. Moreover, addition of inulin, and perhaps other soluble fibers to processed foods, including calorically-rich obesogenic foods, may be a means to ameliorate their detrimental effects.

## GRANTS

This work was supported by National Institute of Diabetes and Digestive and Kidney Diseases Grants DK099071 and DK083890. B. Chassaing is a recipient of the Research Fellowship award from the Crohn's and Colitis Foundation of America.

## DISCLOSURES

No conflicts of interest, financial or otherwise, are declared by the author(s).

## AUTHOR CONTRIBUTIONS

Author contributions: B.C., J.P.M.-B., M.P., E.U., M.R., A.D.P., M.V.-K., and A.T.G. conception and design of research; B.C., J.P.M.-B., and L.Z. performed experiments; B.C. and A.T.G. analyzed data; B.C., J.P.M.-B., M.P., E.U., M.R., A.D.P., M.V.-K., and A.T.G. interpreted results of experiments; B.C. prepared figures; B.C. and A.T.G. drafted manuscript; B.C., J.P.M.-B., M.P., E.U., M.R., A.D.P., M.V.-K., and A.T.G. edited and revised manuscript; B.C., J.P.M.-B., M.P., E.U., M.R., L.Z., A.D.P., M.V.-K., and A.T.G. approved final version of manuscript.
